# ClC-1 Chloride Channel: Inputs on the Structure–Function Relationship of Myotonia Congenita-Causing Mutations

**DOI:** 10.3390/biomedicines11102622

**Published:** 2023-09-24

**Authors:** Oscar Brenes, Michael Pusch, Fernando Morales

**Affiliations:** 1 Departamento de Fisiología, Escuela de Medicina, Universidad de Costa Rica, San José 11501-2060, Costa Rica; oscar.brenes_g@ucr.ac.cr; 2 Centro de Investigación en Neurociencias (CIN), Universidad de Costa Rica, San José 11501-2060, Costa Rica; 3 Istituto di Biofisica, Consiglio Nazionale delle Ricerche (CNR), Via De Marini 6, 16149 Genova, Italy; 4 Instituto de Investigaciones en Salud (INISA), Universidad de Costa Rica, San José 11501-2060, Costa Rica

**Keywords:** myotonia, chloride channel, electrophysiology, channelopathy, mutation

## Abstract

Myotonia congenita is a hereditary muscle disease mainly characterized by muscle hyperexcitability, which leads to a sustained burst of discharges that correlates with the magnitude and duration of involuntary aftercontractions, muscle stiffness, and hypertrophy. Mutations in the chloride voltage-gated channel 1 (*CLCN1*) gene that encodes the skeletal muscle chloride channel (ClC-1) are responsible for this disease, which is commonly known as myotonic chloride channelopathy. The biophysical properties of the mutated channel have been explored and analyzed through in vitro approaches, providing important clues to the general function/dysfunction of the wild-type and mutated channels. After an exhaustive search for *CLCN1* mutations, we report in this review more than 350 different mutations identified in the literature. We start discussing the physiological role of the ClC-1 channel in skeletal muscle functioning. Then, using the reported functional effects of the naturally occurring mutations, we describe the biophysical and structural characteristics of the ClC-1 channel to update the knowledge of the function of each of the ClC-1 helices, and finally, we attempt to point out some patterns regarding the effects of mutations in the different helices and loops of the protein.

## 1. Introduction

Myotonic conditions are hereditary skeletal muscle diseases mainly characterized by muscle hyperexcitability, which leads to a sustained burst of discharges that correlates with the magnitude and duration of involuntary aftercontractions [1,2], a sign known as myotonia. Electrical hyperexcitability is the primary alteration that underlies myotonia and causes a delay in muscle relaxation after muscle action. Most patients affected by these conditions show both electrical (detected in the electromyography) and clinical myotonia [3,4]. Myotonia is the hallmark of several human inherited diseases commonly grouped into 1—non-dystrophic myotonias (NDM), as they show a non-dystrophic muscle phenotype [5]; 2—myotonic dystrophies, either type 1 (DM1) or type 2 (DM2), both showing a highly variable clinical and dystrophic phenotype [6] and are not covered in this review. In the NDM group, there are two main human conditions whose clinical presentation includes myotonia: 1—the sodium channel myotonias that include several disorders with dominant inheritance and are caused by mutations in the sodium voltage-gated channel alpha subunit 4 (*SCN4A*) gene, which encodes the skeletal muscle voltage-gated sodium channel Na_V_4.1 [1]; 2—the chloride channel myotonias that includes the myotonia congenita (MC), which can be either dominantly (Thomsen’s disease, DMC) or recessively (Becker generalized myotonia, RMC) inherited, and caused by mutations in the chloride voltage-gated channel 1 (*CLCN1*) gene that encodes the skeletal muscle chloride channel (ClC-1) [7,8]. Therefore, they are commonly known as myotonic channelopathies, and this review will be focused only on chloride channelopathies.

Mutations in the *CLCN1* gene result in reduced Cl^−^ conductance in skeletal muscle, which is the primary cause of MC. This channelopathy is characterized by muscle hyperexcitability, muscle stiffness, and hypertrophy [9,10]. The clinical picture depends on whether the disease is present in the dominant or the recessive form. The latter is more common and clinically more severe [11]. The two disorders differ by age-at-onset (in infancy or childhood and earlier in DMC), spreading of the myotonia, and a typical transient muscular weakness only present in the recessive trait [7,12,13].

The severity of myotonia in MC is clinically highly variable, ranging from myotonic discharges only detectable during an electromyography test (electrical myotonia) to disabling muscle stiffness at an early age (clinical myotonia) [11]. In MC, almost every skeletal muscle in the body might show muscular stiffness, which can be ameliorated by exercise (warm-up phenomenon) [14]. In addition, MC can be associated with transient weakness during quick movements, lasting only seconds or as long as thirty minutes in Becker’s disease [12].

The *CLCN1* gene has 23 exons and is located on chromosome 7q35. It has been reported that there are roughly more than 200 mutations identified in the gene “http://www.hgmd.cf.ac.uk/ac/index.php (accessed on 30 April 2023)”. However, our exhaustive search (which could be incomplete, since the literature review covers from 1992 to April 2023) for *CLCN1* mutations led us to gather that there are more than 350 different mutations identified in this gene (the specific 364 mutations are summarized in Supplementary Table S1). Although the pathogenesis of MC is not yet fully understood, it is well known that mutations in *CLCN1* produce a reduction in the chloride conductance that leads to membrane hyperexcitability, triggering myotonia [3,4,12].

The biophysical properties of the mutated channel have been explored and analyzed through in vitro approaches, providing important clues to the general function/dysfunction of the wild-type and mutated channels. Despite this, functional analyses have only been carried out for less than half of the mutations identified in the *CLCN1* gene, making it impossible to have a clear and robust genotype–function–structure–phenotype relationship in MC [2]. However, thanks to the recent determination of the ClC-1 structure by cryogenic electron microscopy and atomic modeling to a resolution of 3.36 Å [15,16], a better understanding of the genotype–function–structure–phenotype relationship could be achieved in the coming years [16].

Even before the full ClC-1 structure was described, several studies had already tried to implement new approaches in order to analyze the effect of the mutation on the structure of the channel and, therefore, its dysfunction [17,18,19,20,21,22,23]. Approaches such as homology models, molecular dynamics, or the analysis of structural changes (by other means) caused by *CLCN1* mutations will cope with the improvement in the structure–function relationships in MC and will provide a better explanation of the effect of some mutations on the function of the channel, especially for those mutations that do not seem to differ functionally from the wild-type, show a dual inheritance pattern, or trigger an unusual MC phenotype.

In this review, we aim to address the relationship of different mutations with channel function and not with the clinical phenotype nor with the inheritance pattern (recessive-dominant). We start discussing the physiological role of ClC-1 in skeletal muscle functioning. Then, we describe the biophysical and structural characteristics of the ClC-1 channel in order to update the knowledge of the topological structure of the channel monomers, the amino acids that lined each segment, and the function of each of the ClC-1 helices and loops by using the reported functional effects of the naturally occurring mutations that have been described. Finally, we attempt to point out some patterns regarding the effects of mutations in the different helices and loops of the protein.

## 2. Relevance of the ClC-1 Channel in Skeletal Muscle Physiology

**Figure 1:**
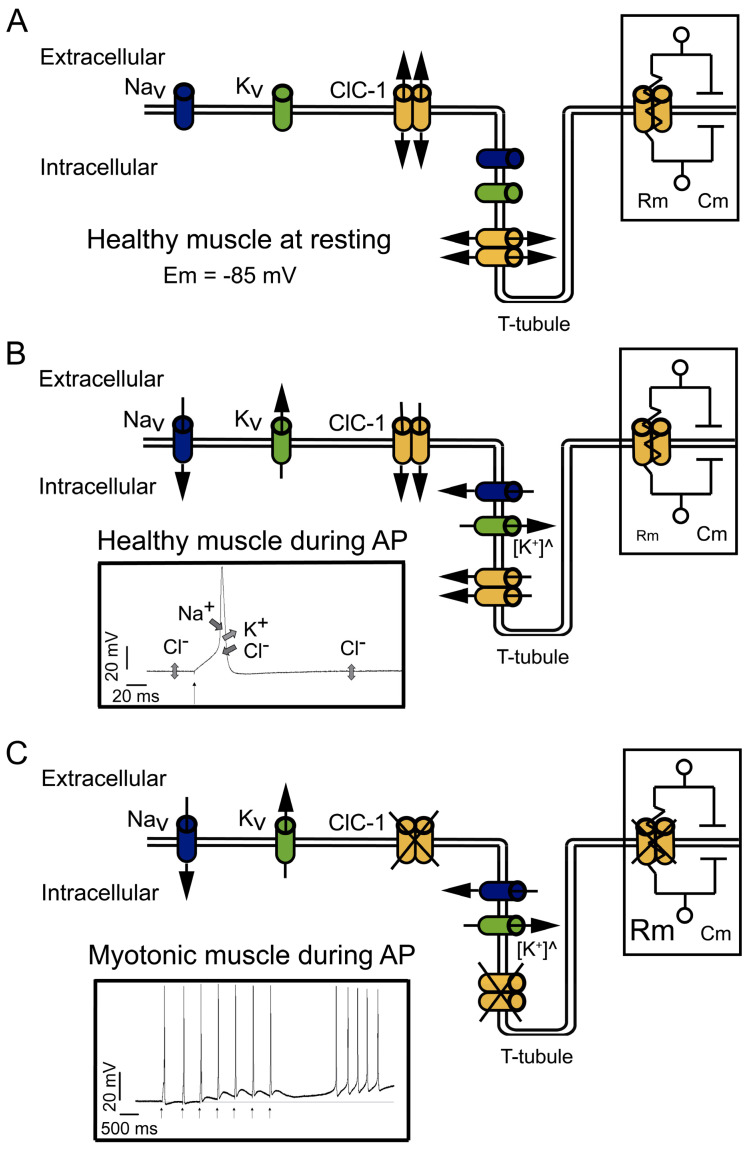
Physiological functions and effects of dysfunction of ClC-1. (**A**) At physiological resting conditions, ClC-1 doubled-barreled channels dominate sarcolemmal conductance, stabilizing the resting membrane potential (Em) at −85 mV. The right insert shows part of the equivalent circuit model of the plasma membrane; the membrane resistance (Rm) is directly dependent on the open ClC-1 channels, and the membrane capacitance (Cm) is dependent on the membrane area. Potassium (K^+^) “leak” channels, opened at rest, are not depicted. (**B**) At physiological conditions, during action potential (AP) firing, sodium (Na^+^) influx through voltage-dependent Na^+^ channels (Na_V_) drive the upstroke of the action potential. Repolarization occurs through K^+^ efflux through voltage-dependent K^+^ channels (K_V_) and the influx of Cl^−^ through ClC-1. Upon repetitive action potential firing, K^+^ slowly accumulates in T-tubules ([K^+^]^^^), causing transient changes in the K^+^ equilibrium potential at a level that can lead to spontaneous (i.e., not neurotransmitter triggered) action potential firing and eventually to inactivation of sodium channels. The contribution of ClC-1 to action potential repolarization reduces K^+^ accumulation effects. In addition, K^+^ building up in T-tubules can induce a depolarization in Em able to spread through the sarcolemma; however, the increased opening of ClC-1 channels decreases Rm (right insert), which in turn decreases the length constant (λ), hampering the spreading of Em changes. The left insert shows the contribution and direction of Na_V_, K_V,_ and ClC-1 current during action potentials; K^+^ “leak” channels’ contribution to Em is not shown. (**C**) In myotonic muscles with dysfunctional ClC-1, during action potential firing, Cl^−^ contribution to the repolarization and stabilization of Em is lost, causing a larger depolarization during K^+^ accumulation in T-tubules. The increased Rm (right insert) helps to propagate the depolarization through the sarcolemma. The left insert shows the action potential induced by a stimulus (black arrows) during voluntary contractions and action potentials spontaneously triggered after voluntary contractions.

Like most excitable cells, human skeletal muscle cells exhibit a quite large negative electrical potential across the membrane in the absence of stimulating signals, which is called resting membrane potential (E_m_). The negative value of E_m_ is related to the high K^+^ conductance, mostly due to the presence in the membrane of the K^+^ channel from subfamilies like the K^+^ leak 2-P-domain (K2P) [24,25]. However, in the adult muscle cells, both the K2P and the inward rectifier K^+^ channels (K_ir_) contribute to E_m_ [26,27]. In addition, muscle cells are unique because of the significant contribution of Cl^−^ to the E_m_ stability, mainly due to a Cl^−^ conductance four-fold larger than K^+^ conductance [28]. Cl^−^ conductance is about 80% of the total resting membrane conductance thanks to the robust expression of the ClC-1 channel [1,29]. ClC-1 is a doubled-barreled pore channel with an open probability of about 20–40% at the resting potential [1,30]. Interestingly, although the Cl^−^ conductance is larger than the K^+^ conductance at resting conditions, K^+^ is the principal factor setting the resting potential, because K^+^ ions are actively imported by the Na-K-ATPase, while the Cl^−^ concentration is “passively” adjusted according to the negative membrane potential. This leads to the fact that the intracellular Cl^−^ concentration is low and that the Cl^−^ equilibrium potential is very close to E_m_ (about −85 mV) [31]. The large Cl^−^ conductance thereby stabilizes E_m_ close to the K^+^ equilibrium potential (Figure 1A).

Cl^−^ currents become even more significant during muscle activity (Figure 1B). During stimulation, the influx of Na^+^ through the voltage-gated 1.4 sodium channel (Na_V_1.4) is responsible for driving the electrical potential away from its resting value toward positive values, a phase called depolarization (left insert in Figure 1B). The subsequent repolarization phase of the action potential is usually due to the inactivation of Na_V_1.4 channels in conjunction with the opening of voltage-gated delayed rectifier K^+^ channels [27,32]. In addition to these two classical mechanisms, skeletal muscle is unique among excitable tissues because the contribution from Cl^−^ influx rivals that of K^+^ outflux during the repolarization. ClC-1 open probability increases with depolarization [1,30], and with membrane potentials that can reach even positive values, the electromotive force for Cl^−^ increases and, consequently, increases the Cl^−^ currents, contributing to repolarization (left insert in Figure 1B). In case of a complete lack of ClC-1, as in recessive myotonia with loss-of-function mutations, the K^+^ conductance is sufficient to maintain a negative resting potential and to impede spontaneous action potential firing, but is not sufficient to impede repetitive firing after a single nerve impulse.

The currents through ClC-1 channels are believed to be especially important in the muscular transverse tubule system called T-tubules [17,33]. K^+^ efflux could be enough to control the repolarization phase of the action potentials on the surface of the cells (Figure 1B), but not in the T-tubules, which are a narrow reticular network with tubules having a diameter of about 40 nm. The network extends across a 30 µm fiber radius with a high surface area/volume ratio (equivalent to 106 cm^2^/mL). This narrow space limits the equilibrium of ions by simple diffusion between its lumen and the surrounding interstitial fluid. Because of the narrow space and limited diffusion, during muscle action potentials, outward K^+^ currents result in a substantial increase in T-tubular K^+^ concentration (Figure 1B). The impairment in the concentration gradient across the membrane, and therefore the decrease in the electrochemical driving force that drives the K^+^ efflux, decreases the K^+^ capacity to repolarize the cell. The increased opening of ClC-1 channels and increased driving force allow a Cl^−^ influx that contributes to the repolarization, thereby reducing the requirements for K^+^ efflux in muscle cells (Figure 1B) [1,28,34].

Moreover, during high-frequency stimulation, it has been shown in cultured muscle cells that in the absence of Cl^−^, the accumulation of K^+^ can induce depolarization of about 1 mV for each action potential. Therefore, after a few spikes, the depolarization shift in the resting membrane potential is enough to create a depolarization-driven series of after-discharges of self-sustained action potential (a phenomenon called myotonic run), characteristic of myotonic muscles [33] (left insert in Figure 1C). In healthy muscle cells, the high Cl^−^ conductance opposes the depolarization induced by the K^+^ accumulation in the tubular system, suppressing the possible hyperexcitability of the muscle.

In myotonic muscles, the hyperexcitability can be counteracted by the warm-up phenomenon, and the hypotheses explaining this phenomenon are related to an activity-dependent reduction in cellular pH and the depolarization induced by K^+^ accumulation. The proposed mechanisms implying K^+^ accumulation in T-tubules during activity imply that the depolarization induced can be sufficiently large enough to impair sodium channel recovery after inactivation, producing the accumulation of inactive Na_V_1.4 channels and reducing cellular excitability and myotonic runs [1].

In addition, during intense muscle activity, if the K^+^ accumulation in the T-tubules induces depolarization of the resting potential, this depolarization can spread along the sarcolemma. However, the presence of ClC-1 channels and the high Cl^−^ conductance of skeletal muscle reduces the membrane resistance (R_m_) (right insert in Figure 1B). Small R_m_ reduces sarcolemmal length constant (λ) and prevents the local propagation of depolarizations [28]. However, in the absence of functional ClC-1 channels, it is plausible that the increased R_m_ (right insert in Figure 1C) favors depolarization spreading.

Altogether, the role of Cl^−^ currents in muscle electrophysiological behavior can be summarized in two stages. First, during inactivity, the large chloride conductance contributes to the stabilization of the resting membrane potential. Second, during stimulation, Cl^−^ conductance counteracts the change in the voltage, increasing the current threshold needed to elicit an action potential, prolonging the time course of the electrotonic depolarization of the pre-potential toward the action potential threshold, allowing the correct repolarization of the muscle fiber during the action potential firing and preventing the depolarization induced by K^+^ accumulation locally in T-tubules and through sarcolemma.

In conclusion, the ClC-1 channel can be considered a stabilizer of the resting membrane potential and a suppressor of repetitive action potentials [1,28,33], and its malfunction can be related to increased muscle excitability.

Interestingly, there is an inverse relationship between ClC-1 channel activity and muscle fatigue, a sign observed in NDM patients [35]. During prolonged muscle activity, acidification of the cell and calcium-dependent activation of PKC occurs, and in vitro studies have shown that both low pH and PKC activation lead to ClC-1 inhibition [36,37]. Consequently, it could be expected that ClC-1 inhibition induces an increase in membrane electrical resistance and an increase in cellular excitability, preserving muscle activity and mediating muscle adaptation to intense exercise. However, prolonged contractions can drive the reduction of the ATP concentration due to hydrolysis by myosin and ion pumps, which can induce the activation of K_ATP_ channels and unblock the ATP inhibition of ClC-1, resulting in higher activity of the chloride channel and the reduction of action potential amplitude and cell excitability, processes that are related with muscle fatigue [30,36].

## 3. ClC-1 Localization Controversy: T-Tubule System and/or Sarcolemma

Several studies have been carried out to identify the exact localization of ClC-1, but thus far, there is no consensus among research groups. The controversy started many years ago when, with elegant experiments, Hodgkin and Horowicz [38] indicated for the first time that the muscle chloride conductance was localized in the sarcolemma, findings that were also found by Gurnett et al. in 1995 [39]. However, in the late 70s, two independent studies showed evidence that suggested that the Cl^−^ conductance was mainly localized in the T-tubule system [40,41], data that were confirmed in more recent investigations [42,43].

Interestingly, depending on the assay used to identify its location, the location appeared to be different. For instance, Papponen et al., 2005 [44] localized ClC-1 in the interfibrillar spaces from muscle cryosections with prominent sarcolemmal localization in a non-uniform way, being present even in the neuromuscular junction. However, when fibers were isolated and cultured in vitro, the ClC-1 was absent at the sarcolemmal level [44]. Thus, Papponen’s group proposed that the isolation process of myofibers and the resultant denervation of the myofibers changed the distribution pattern of ClC-1, explaining the lack of ClC-1 in the sarcolemma [44].

In 2010, in a series of fancy experiments using dissociated muscle fibers, Lueck et al. [45] showed strong evidence that functional ClC-1 channels are localized exclusively within the sarcolemma. However, in 2011, DiFranco et al. [46] measured simultaneously muscle fiber Cl^−^ currents by the two-electrode voltage clamp method and local T-tubule Cl^−^ currents by potentiometric optical indicators and suggested that ClC-1 is present at both the sarcolemmal and T-tubules membrane compartments. However, ClC-1 channel distribution is still an open question, and the controversy remains [47].

## 4. ClC-1 Structure–Function Relationship Overview

**Figure 2:**
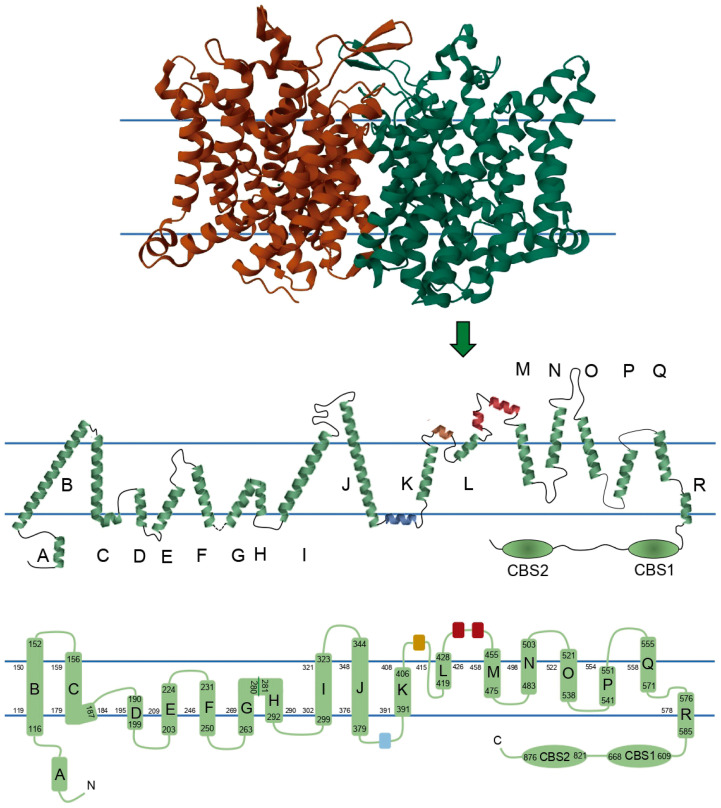
Two-dimensional topology of ClC-1 with schematic representations of a ClC-1 monomer. Upper panel shows each ClC-1 monomer with different color. In the central panel, the size and orientation of each helix, loop, and helical stretch are based on the 3D structure described in the Protein Data Bank under the entry ID 6COY, its membrane topology, segment localizations, and secondary structure. Monomers are composed of seventeen helices embedded in the membrane, while two helices and the two CBS domains are intracellular, and three helices are extracellular. Blue parallel lines in central and bottom panels represent the position of the membrane. In central and bottom panels the blue, brown, and red segments represent the helical stretch segments identified in human ClC-1, and green segments represent the helices classically reported in the literature with the nomenclature A to R and the CBS domains. Bottom panel shows a 2D topology where the α-helices are drawn as cylinders, the numbers limiting the cylinders represent the amino acids that enclose helices B to R and CBS segments, and the numbers lining each membrane monolayer represent the amino acids that limit each intramembrane segment.

ClC-1 is composed of two identical subunits of 988 amino acid residues. Traditionally, it has been thought that each of the monomers consists of 18 α-helices: 17 α-helices expanding the membrane, named from B to R, 1 intracellular N-terminal helix named A, and 2 intracellular C-terminal cystathionin-β-synthase (CBS) segments. However, recent structures showed some subtle differences. In order to show these differences, we reconstructed a 2D topology of the channel based on the 3D structure published by Park and MacKinnon (2018) (Figure 2) [15]. Some of the biggest differences are 1—the absence of a loop connecting helices G and H, where instead there is a torsion in the helix; 2—the linker between helix J and K includes a small helix (colored blue in Figure 2) running parallel to the membrane, apparently being intracellular or in close association with the intracellular side of the membrane; 3—in a similar way, the linker between K and L harbors a small extracellular helix (colored brown in Figure 2), and additionally, this region exits and reenters the membrane before the beginning of helix L; and 4—there are two small helices between the helices L and M (colored red in Figure 2). Because of differences 2, 3, and 4, we propose calling the segments between helices J-K, K-L, and L-M as helical stretch J-K, helical stretch K-L, and helical stretch L-M, respectively, instead of loops.

Based on the 3D structure, we added to the 2D topology the amino acid numbers that enclose α-helices B to R and CBS segments, and lining the membrane, we added the amino acid numbers that limited each intramembrane segment of the channel, allowing in this way the localization of each of the 364 reported mutations (Supplementary Table S1) and furnishing a simple way to localize future mutations.

The functions of these regions have been studied through the analysis of naturally occurring mutations in different patients suffering from myotonic disorders. However, due to the lack of information on most *CLCN1* mutations, on topics such as 1—functional effects on the channel; 2—structural effect on the channel; 3—description or identification of all functional segments of the channel; and 4—the lack of accurate genotype–phenotype correlations in congenital myotonia [2], it has been extremely difficult to relate the available data with the whole MC clinical picture and to establish proper structure–function relationships in the ClC-1 channel, which are vague at this moment. A better understanding of these relationships will undoubtedly provide a better knowledge of congenital myotonia. Thus, the information from the functional data of the 165 mutations described in the literature (Supplementary Table S2) is summarized in Table 1, and in the next sections, we describe some hints on these relationships based on the available data in the literature.

### 4.1. Channel Disruption

A possible modification affecting channel function in muscle physiology is the truncation of the protein. At least 37 mutations have been reported that cause premature stop codons, occurring as early as in amino acid 33 (Y33*) or as late as in amino acid 976 (R976*). However, just three of them have been analyzed functionally. The most N-terminal one is W322*, which was described in Costa Rican patients diagnosed with Thomsen’s disease or Becker’s myotonia. Expression of this mutant in *Xenopus* oocytes showed a complete loss of function. It was very likely that the mutation led to a truncated, not functional protein, lacking 2/3 of the total sequence, or led to a nonsense-mediated mRNA decay [18].

Based on this, it should be expected that all stop codons occurring between amino acids Y33 and W322 (see Supplementary Table S1) will also lead to non-functional proteins and possibly RNA decay. Most of these mutants have a recessive inheritance in agreement with the general hypothesis that the loss of one *CLCN1* allele is not sufficient to cause myotonia [48].

Between W322 and Y686, there are nine stop codon mutants (see Supplementary Table S1) that have not been functionally described, but that probably also induce non-functional proteins. This is supported by the fact that the Y686* expressed in HEK293 cells did not generate ClC-1-related currents [49]. It would be interesting to functionally analyze stop codon-forming mutations beyond Y686*, as this could provide evidence of the location in which the protein starts being functional. A clue about this is provided by the mutation Q788*: although it has not been functionally characterized, it was described that it behaves in a dominant manner [50], likely due to a dominant-negative effect, where the full ClC-1 structure is modified by the interaction of wild-type ClC-1 and an altered ClC-1 structure, affecting thereby the chloride conductance in the patients.

The last characterized mutant with an early stop codon is R894*, which lacks the C-terminal distal to the second CBS segment. When this truncated protein is expressed in human kidney cells (tsA201), it can produce functional channels, but with smaller current amplitudes than wild-type (WT) channels [51]. Macías et al. [52] reported a reduced surface expression of 10% for this mutant in *Xenopus* oocytes, but this result was not confirmed in muscle cells [53]. Therefore, the absence of the channel in the membrane does not seem to be the cause of the decreased currents. Interestingly, Hebeisen and Fahlke [54] reported currents similar to WT for this mutation, with a negative shift in the open probability (increasing open probability at physiological voltages) for the naturally occurring R894* and a positive shift of the induced K875*, both related to altered anion binding and probably structural changes that affect the outer vestibule of the conduction pathway [54].

Based on these results, it is plausible to suggest that the last C-terminal loop is involved in voltage-dependence regulation with both positive and negative shifts, ion pathway structure, and probably trafficking (Table 1).

### 4.2. Membrane Localization

In addition to the channel’s biophysical function per se, protein folding efficiency, early posttranslational modification, and protein conformational stability are key aspects determining the trafficking, membrane localization, and, therefore, the physiological function of the channel. This is underlined by the fact that at least twelve different mutations affecting ClC-1 abundance at the plasma membrane have been associated with MC (Supplementary Table S2) [49,51,53,54,55,56,57,58,59,60]. It is not clear which ClC-1 segments are related to settling the fate of nascent ClC-1 protein and how they can affect channel membrane localization, but it has been suggested that the CBS segments are crucially involved in this [19]. CBS role cannot be supported by naturally occurring mutations since none of the twelve mutations identified on these segments (and studied functionally) were tested for membrane localization properties. On the other hand, the analysis of naturally occurring mutations points to two additional protein structures that are very likely related to protein stability. Three missense mutations located in one of the extracellular helices in the helical stretch K-L and two other mutations located at the C-terminal region after the CBS2 segment have been described to reduce membrane localization, possibly due to folding defects [51,54,55,58,59,60,61].

A total of 23 mutations have been reported in the helical stretch L-M, and 12 have been reported in the last C-terminal loop (Supplementary Table S1); six of them were reported with smaller or complete loss of currents that could be related to misfolding. It is important that future reports of mutations in these segments test membrane localization and biochemical stability. Interestingly, G898R mutation was reported with current amplitude and open probability similar to the WT channel when analyzed in *Xenopus* oocytes. In this case, it could be useful to test membrane localization or protein stability, specifically in muscle cell lines. Supporting this function, the A885P mutation in the C-terminal region of the goat ClC-1 channel also seems to affect membrane localization [52,62].

Interestingly, the three mutations in the helical stretch L-M involve substitutions of hydrophobic for hydrophilic amino acid, and since this segment is located extracellularly (Figure 2), it is possible that the substitutions provoke structural changes in the helix, affecting protein interaction during trafficking or interactions with the extracellular matrix. The exact role of the helical stretch L-M and the C-terminal region in channel localization must be studied in the future; nevertheless, these studies should be taken with caution since, at least for the R894* mutant, a trafficking defect was reported in *Xenopus* oocytes, but it was normal in muscle cells [52,53]. The rest of the mutants mentioned here were tested in *Xenopus* oocytes or HEK293 cells (Supplementary Table S2); therefore, in order to obtain more valid results, it is recommended to analyze membrane localization in muscle cells.

### 4.3. Pore Properties

Each subunit of the ClC-1 channel forms its own Cl^−^-conducting pore, with a minimum diameter estimated at around 4.5 Å and an electropositive surface in its extracellular face [16,30]. Each Cl^−^ pathway has three serial Cl^−^ binding sites, named S_ext_, S_cen_, and S_int_, for external (near extracellular compartment), central, and internal (near intracellular compartment), respectively. Interestingly, the pore pathway bifurcates on the intracellular side, one following the canonical Cl^−^ pathway found in other ClC proteins and the other putatively being a secondary pathway directed toward the protomer–protomer boundary on the cytosolic surface [15]. However, the relevance of this pathway is still obscure.

It has been pointed out that the D, F, N, and R membrane helices contribute to the Cl^−^ pathway, and among them, D, F, and N form the Cl^−^ selectivity filter. Specifically, helices N (F484 and M485) and F (from G230 to G233, including the conserved Glu gate E232) are arranged to coordinate Cl^−^ on S_ext_. The Cl^−^ in S_cen_ interacts with Y578 of helix R and with S189 of the C-D loop (just prior to the beginning of helix D). Finally, the Cl^−^ in S_int_ seems to interact with amino acids around L577 and I581 of helix R [15].

In this regard, nine different mutations have been described to affect ion selectivity, i.e., directly modifying the pore pathway. Three of these mutations are located in helix D [63,64], changing negative or non-polar for positive or polar amino acids. Thus, similar mutations, like L198H located in helix D, could also affect ion selectivity. We suggest that this kind of mutation should be tested for channel ion selectivity in the future.

Also, mutations in helices C [64], G [65], and I and in the M-N loop [66,67] have been reported to alter ion selectivity (Supplementary Table S2). Nonetheless, more studies are necessary to clarify the role of helices C, G, and I and the M-N loop as part of the Cl^−^ selectivity filter or if mutations indirectly alter its structure, for example, by analyzing mutations like G160H or C179W in helix C more deeply. In addition, two mutations were reported affecting S189, two for G230, two for G233, one for F484, and two for M485, all contributing to the Cl^−^-binding sites, and some of them presented smaller currents. In the future, these or other mutations in these amino acids should be tested for specific effects on ion selectivity.

The ClC-1 single-channel conductance has been reported to be around 1 pS at voltages more negative than −100 mV [68,69]. For ten mutations located in the N-terminal, in helices B, C, G, and N, and in the D-E loop and helical stretch K-L, the single-channel conductance has been measured (Supplementary Table S2). Most of them showed normal conductance, but two mutations (M485V in helix N and C277Y in helix G) resulted in a smaller single-channel conductance [65,70]. This could be expected for helix N, which contributes to the Cl^−^ pathway, and residue M485, which appears important for pore structure and is one of the Cl^−^ binding sites, but not for helix G, supporting its possible role in the pore pathway functioning.

### 4.4. Gating

In the ClC-1 channel, as in other ClC family members, two types of gates have been described: two independent protopore gates that control each subunit independently and one common gate for the two subunits [71]. Each of the gates has its own opening processes, and both are voltage-dependent [72]. The open probability of ClC-1 channels and their voltage dependence can be analyzed from macroscopic currents, but to allow Cl^−^ movement, both ClC-1 channel gates must be opened. However, analysis can go further through protocols that allow the separation of independent and common gate open probabilities [72]. Different authors in the literature have chosen different paths; therefore, the available information on the effects of each mutation in ClC-1 open probability or the effect on specific gates is fragmented.

**Figure 3:**
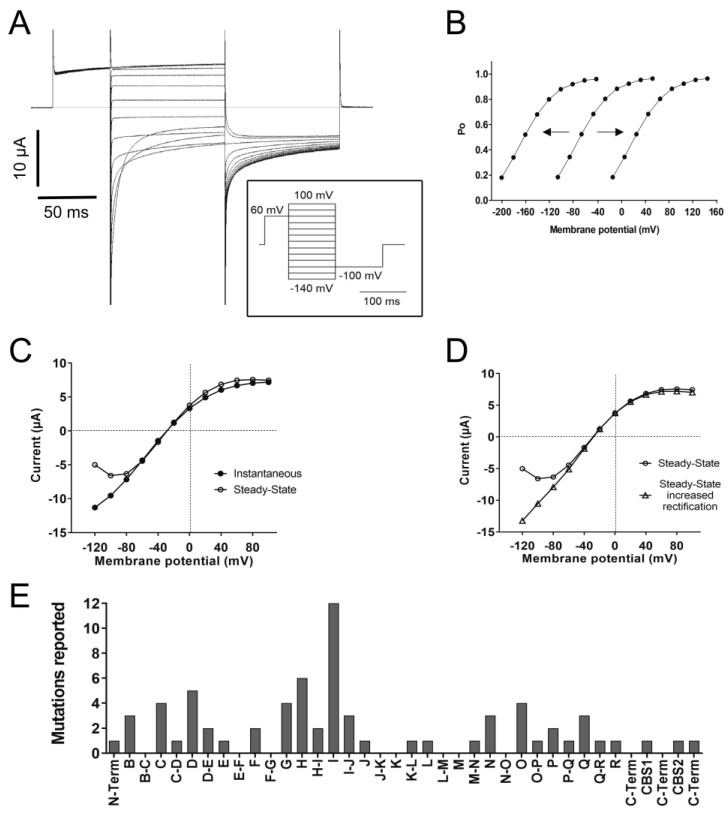
Functional characteristics of ClC-1. (**A**) The left panel shows a representative voltage-clamp recording of ClC-1 currents elicited by the stimulation protocol shown in the insert. Note the increase in currents with depolarization and the deactivation upon hyperpolarization. (**B**) The apparent open probability (Po) is estimated by measuring the initial current at the constant “tail” voltage of −100 mV [I(V)] as a function of the test potential V (varying between −140 and +100 mV, insert in 3A) and calculated by the normalization Po = I(V)/I_max_, where I_max_ is the maximal tail current. Note that the expected Po with ~40% probability around muscle resting potential (−85 mV) in the central line (WT condition). Hypothetical “shifts” of Po to the left (i.e., to more negative voltages) or to the right (i.e., to more positive voltages) are also shown. (**C**) Current–voltage (I-V) relationship of the “instantaneous currents” measured at the beginning of the pulse step and the steady-state current measured towards the end of the step. (**D**) I-V relationship of the steady-state currents and an example of mutant current with increased inward rectification. (**E**) Frequency histogram of reported mutations inducing a positive shift in the voltage-dependence of open probability according to the different helices and loops (from Supplementary Table S2). All the data and graphs were obtained in our laboratory.

As mentioned before, ClC-1 is a channel with an open probability of about 20–40% at muscle resting potential. The open probability is increased by depolarization and decreased by hyperpolarization, even though it never closes completely (Figure 3A,B) [1,30,72]. In voltage-dependent cation channels, a transmembrane segment containing positively charged Arg and Lys residues plays the role of a voltage-sensing domain, which is independent of the pore domain and the ion movement through the pore pathway [73]. No such domain is present in ClC channels; in contrast, the gating process of the two ClC-1 protopores is strongly coupled to the anion movements in the pore [74,75]. The charged side chain of the conserved glutamate called “gating glutamate” (Glu gate, E232) in helix F protrudes into the ion transport pathway where it occupies the S_ext_ anion-binding site, preventing both Cl^−^ binding and flow. Changes in the membrane potential modulate channel opening in such a way that depolarization favors Cl^−^ binding instead of E232, allowing conduction, whereas hyperpolarization favors E232 binding and channel closure [61,74].

Since Cl^−^ interaction with S_ext_ is part of the gating process, gating is modified by the Cl^−^ concentration. In particular, a reduction in the extracellular Cl^−^ concentration leads to a positive shift in the voltage dependence of the open probability, hampering the opening, while changes in intracellular Cl^−^ concentration have almost no effect [30]. In agreement with the tight coupling of permeation and gating, mutations in the helices that contribute to the ClC-1 ion pathway (D, F, N, and R) or are involved in anion binding (as part of S_ext_, S_cen_, and S_int_) affect voltage dependence and gating [15,19,21,30].

At least 74 mutations (Supplementary Table S2) have been reported to affect the open probability with positive shifts, negative shifts (Figure 3B), or even inversions in the voltage dependence. The most common effect (thus far) reported is a positive shift (at least 69 mutations). A positive shift in the voltage dependence of the open probability means that the channel responsiveness to voltage changes decreased; therefore, stronger depolarizations are needed to increase the open probability. A frequency histogram of these mutants pointed to three main positions in the protein related to this behavior (Figure 3E): the first around helix I, the second towards helix D, and the third and smaller one around helices N, O, P, and Q.

Interestingly, more than half of the mutations found around helix I were substitutions to polar hydrophilic amino acids, most of them from aromatic or non-polar hydrophobic residues. Mutations without functional characterization, like Y302C or G305E, and future new mutations with this kind of substitution could be predicted to induce positive shifts in voltage dependence, and helix I should be studied deeply about its contribution to Cl^−^ binding and flow.

Regarding each of the gates, it has been shown that at negative voltages (more negative than −50 mV), the kinetics of the independent and common gates are similar, but at more positive voltages, the time constants drift apart progressively, such that they have been called fast and slow gates [72,74]. The fast gate controls the opening and closing of the independent subunits, while the slow gate controls the opening and closing of the two subunits simultaneously (common gate), both becoming faster when the voltage becomes more positive [72].

The fast gate has been related to the conserved gating glutamate (E232) in helix F, where the mutation G230E has been shown to abolish currents or trigger slower and less complete deactivation by hyperpolarization and even a change in E_rev_, suggesting affected anion selectivity (Supplementary Table S2). The identity of the slow gate remains elusive, but since the slow gate is a common gate for both protopores, it has been suggested that the helices that form the interface between monomers (H, I, P, and Q) could be part of the slow gate, in addition to the CBS and C-terminal region as modulators through interactions with transmembrane structures [16].

Several mutations affect the open probability of both the fast and slow gates (Supplementary Table S2), and they are distributed throughout the channel sequence with no pattern. However, eight mutations evoked open probability shifts only in the slow gate; three of them were in helix I, and one was in helix H, supporting their function as part of the slow gate (Table 1). In addition, two mutations on segments CBS1 and C-terminal region induced an inversion in voltage dependency and a negative shift, respectively, supporting the function of these segments as modulators of the slow gate (Table 1).

Interestingly, there are mutants in helix P and M-N and O-P loops that exclusively affect the slow gate. Structural analysis of these mutations or new ones in these regions should be performed to test if these are part of the slow gate or have indirect effects on gate functioning.

The kinetics of activation and/or deactivation of the fast and slow gates can also be affected by mutations. Fast gate activation by depolarization is likely most important during the period of the muscle’s action potential [74]. A characteristic feature of ClC-1 is that it deactivates within about 50 ms with at least two exponential components when the voltage is stepped to values more negative than the normal muscle resting potential (Figure 3A) [68]. The faster time constant of deactivation is primarily associated with almost complete fast gate closure, while the slower component mostly reflects (incomplete) slow gate closure [72].

At least twelve mutations have been reported to induce slower activation at positive voltages (in helices B, D, N, O, and Q, the I-J loop, the C-terminus, and in CBS1, Supplementary Table S2) [21,49,63,64,76,77], which suggests that the contribution of ClC-1 to repolarization during action potentials decreases.

Mutations in most of these helices and segments also affected the deactivation of both fast and slow gates, inducing a slower, less complete, or complete loss of deactivation. Specifically, mutations in helices B, D, N, O, and Q and in the I-J loop affect both activation and deactivation. Since helices D and N are part of the pore pathway and selectivity filters, it is plausible that mutations in these could affect gating kinetics. Therefore, additional studies should be undertaken on mutations in helices B, O, and Q to evaluate how they are affecting these processes.

Furthermore, future research should consider investigating the role of the I-J loop since this loop has already been proposed to play a role in controlling chloride passage, and at least 14 disease-associated mutations have been reported in the literature in this loop. Twelve of these mutations were missense, but only five of them have been functionally studied, with reported effects on activation and deactivation of fast and slow gates, together with changes in channel voltage dependence. Interestingly, most of the mutations are changes in non-polar to polar amino acids (Supplementary Table S2). Thus, the study of naturally occurring mutations in this part of the channel could be useful to better understand the channel kinetics.

Interestingly, three mutations in H-I and P-Q loops and in helix N affected exclusively slow gate deactivation, again supporting the role of loop H-I and helices P and Q in slow gating and suggesting helix N as a possible slow gate modulator (Table 1).

### 4.5. Channel Rectification

Additionally, nine mutations, three of them without a reported positive shift in open probability (Supplementary Table S2) in helices D, G, and Q, have been reported to prevent saturation at positive potentials, hence increasing outward rectification. This suggests a role of these helices in the modulation of saturation, a possibility that should be tested in future studies.

Another characteristic feature of ClC-1 is that, after maximal activation, the instantaneous current–voltage relationship exhibits stronger inward rectification than steady-state currents (Figure 3C). As mentioned before, at least 23 mutations have been reported to completely abolish or decrease the deactivation of the fast and/or the slow gate, an effect that can be related to increased inward rectification (Figure 3D). However, an increase in inward rectification was reported for just six mutations on helices B, N, and O and the H-I loop.

**Figure 4:**
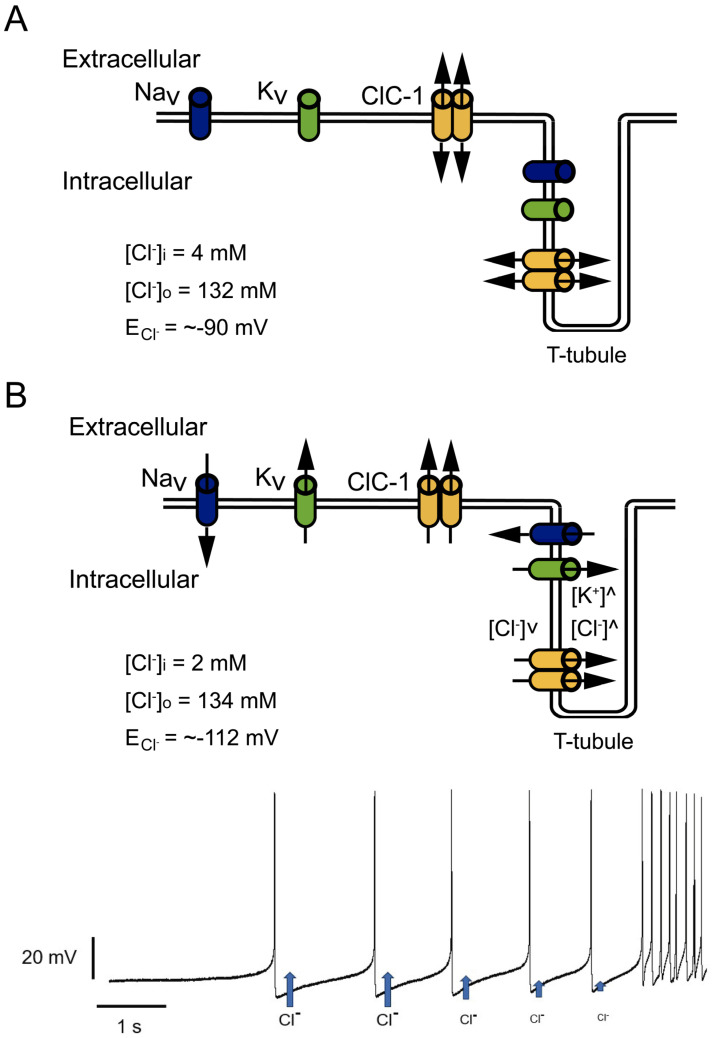
Effect of increased inward but decreased outward currents in Cl^−^ currents. (**A**) At physiological conditions, Cl^−^ inside and outside concentrations allow an equilibrium potential (E_Cl_^−^) close to muscle resting potential, where Cl^−^ influx and outflux play a stabilizing role. (**B**) Mutant channels with pronounced inward rectification allow Cl^−^ outflux during trains of action potentials without influx during action potential repolarization. In this condition, repetitive activity can cause intracellular Cl^−^ depletion and T-tubule Cl^−^ accumulation, progressively decreasing Cl^−^ outflux during repetitive firing (blue arrows getting smaller after each action potential in bottom panel), and shifting E_Cl_^−^ to more negative potentials.

Special attention should be paid to the relationship between increased inward rectification and decreased outward current amplitudes. Mutations such as D136G showed loss of deactivation with hyperpolarization and the absence of outward currents at voltages positive to the Cl^−^ reversal potential [78]. This behavior can allow the Cl^−^ efflux but prevents Cl^−^ influx (Figure 4A). At resting conditions, this behavior should not affect E_m_ since, as mentioned before, E_m_ is principally set by the resting K^+^ conductance. However, in a muscle under repetitive stimulation, a mutant channel with this behavior could cause Cl^−^ depletion and changes in the equilibrium potential of this ion to values much more negative than muscle resting membrane potential (Figure 4A,B), affecting Cl^−^ electromotive force and hampering the stabilizing function of the channel [78].

Another interesting mutation is S132C, in helix B, which triggers an increased opening with hyperpolarization in conditions of high internal Cl^−^ instead of the hyperpolarization-induced deactivation of WT channels [79]. In addition, there are two close mutations (I553F and H555N) on the P-Q loop and helix Q that decreased the inward rectification of instantaneous currents [80]. Clearly, a better understanding of ClC-1 functioning would benefit from future studies of mutations in helix B and around helices N, O, P, and Q that analyze effects on channel inward and outward rectification.

### 4.6. ClC-1 Modulation

As pointed out before, intracellular pH affects ClC-1 gating, triggering faster deactivation at more alkaline pH and channel inhibition by intracellular acidification when studied in the presence of ATP at physiological concentrations [30,36,37]. ATP acts synergistically with acidic pH to inhibit ClC-1 by shifting the voltage dependence of common gating to more positive potentials [36]. Both protons and ATP stabilize the closed state of the common gate and together induce an alteration of the voltage dependence of the closing rate [36].

In this regard, ATP binding site is between the two CBS segments [23], and the structural analysis performed by Wang et al. [16] showed a more rigid CBS segment at lower pH in the presence of nucleotides, but more flexibility at higher pH in the absence of nucleotides. In addition, ClC-1 also has a large loop downstream of the CBS segments, but the function of this loop is poorly understood, although it might be related to PKC-induced inhibition [15,21]. To the best of our knowledge, none of the mutations described in the C-terminal region or in the CBS domains has been tested for possible effects related to ATP, pH, or PKC regulation. This is intriguing, since at least 90 disease-causing mutations have been reported in this region. Therefore, it is important that more research on ClC-1 modulation will be carried out.

### 4.7. Apparently WT-like Channels

Several *CLCN1* variants with strong genetic evidence of causal implication in myotonia have been reported to behave very similarly to WT channels in in vitro electrophysiological studies (Supplementary Table S2). Normally, such variants might be considered non-disease-causing variants, i.e., benign polymorphisms. However, sometimes, the current–voltage relationships and the voltage dependence are the only analyses performed. Deeper analysis must be performed to test, for example, activation and deactivation kinetics, membrane localization, rectification, or ion selectivity.

For example, mutation Y261C displayed current amplitudes similar to WT channels; however, a deeper analysis showed that mutant channels lost the capacity for NAD^+^ interaction and modulation [70,81]. Also, A331S, F333L, and L628P showed normal current amplitude and open probabilities, but the analysis of activation kinetics showed a slower activation with depolarization [21,82]. Similarly, L587V showed current amplitude and open probabilities similar to WT, but faster opening with depolarization and faster and more complete deactivation of the slow gate [82] (Supplementary Table S2). Also, it is important to point out that often, the mutations are analyzed through the expression of the channels on *Xenopus* oocytes or human cell lines unrelated to muscle tissue. It cannot be excluded that in these models, intracellular proteins that interact with ClC-1 are lacking. Of the 26 MC mutations reported in the literature that display a WT-like behavior, 16 are located intracellularly (Supplementary Tables S1 and S2). Thus, it is not possible to rule out the possibility that they are mutations that affect channel interaction with intracellular targets in muscle cells.

## 5. Possible Implications for Channel Pharmacological Modulation

So far, no commercial drugs directly target ClC-1. However, taking into consideration the structure–function relationships revealed by naturally occurring mutations and the role of ClC-1 in MC, it is feasible to suggest some channel segments as targets of future drugs developed to modulate the effect of specific *CLCN1* mutations found in myotonic patients.

For example, in cases of dominant gate-shifting mutations, such as S132C, S189C, I290M, V299L, or F306L, among others, the development of gating corrector molecules might be useful. In particular, molecules might be developed to target the segments related to gating shift mutations, like helix I, helix D, and around helices N, O, P, and Q (Figure 3E). Also, in cases of recessive folding-defective mutants, where membrane trafficking and protein stability are affected, the development of pharmacological chaperones could be helpful. In this matter, several mutations are located in the helical stretch K-L and at the C-terminal region after the CBS2 segment. For these, it might be possible to develop molecules that bind these structures and that could have effects on protein stability and/or trafficking.

The screening of small-molecule correctors that can be useful in the development of drugs targeting specific defects in channels has been conducted for the cystic fibrosis transmembrane conductance regulator (CFTR), an epithelial Cl^−^ channel. It has been possible to identify molecules that correct open channel probability, stabilize the protein fold, facilitate translocation to the cellular membrane, and/or act at several levels [83,84]. In a similar way, carbamazepine has been proposed as a drug able to improve ATP-sensitive K^+^ channel trafficking, a process involved in congenital hyperinsulinism [85].

Finally, since MC is a pathology characterized by increased muscle excitability, sodium channel blockers are generally used to reduce excitability [17]. Also, in diseases associated with muscle hypoexcitability, like myasthenia gravis, inhibitors of ClC-1 have been proposed as possible treatments [86]. Therefore, the screening of molecules capable of inhibiting the channel that controls muscle behavior can contribute to the future development of drugs against several muscle pathologies.

## 6. Conclusions

Even though relatively few *CLCN1* mutations have been functionally investigated, it is possible to cluster the ClC-1 helices and loops based on their putative function, as shown in Table 1. Clustering these functions is important to underscore the necessity of studying the functional effects of more than 40 mutations located in helices with unknown functions, like E, K, L, and M. Also, it is important to embrace a new interpretation of mutation analysis like the “mutational survivorship bias” [87], which suggests that using only visible variants leads to partial conclusion regarding the function and relevance of protein segments and that it is likely to identify important domains that could be of high relevance but low tolerability to mutation. Regions like helix R must be analyzed by additional approaches, such as computational modeling or directed mutagenesis, since the location and structural analysis suggest that it is important for several channel characteristics, but just one mutation has been identified in this helix. However, carrying out additional approaches to obtain more and better hints on the function–structure relationships regarding the ClC-1 channel might be compromised, mainly due to the availability of proper materials, such as human muscle biopsies, or knock-in mouse models, as discussed previously [2]. We hope that this review will help future research to guide specific analysis of undescribed or new ClC-1 mutations found in myotonic patients in order to achieve a better understanding of the functioning of the human ClC-1 channel.

## Figures and Tables

**Table 1:** Updated functions of ClC-1 helices and loops based on effects of naturally occurring mutations.

Domain	Function	Mutations Reported
N-terminal and A	Membrane localization + Voltage-dependence of open probability + Link between the two subunits *	17
B	Membrane localization + Voltage-dependence of fast and slow gates + Channel opening kinetics at positive voltages and deactivation at negative voltages of fast and slow gates + Effects on rectification + Link between the two subunits *	15
B-C loop	No mutations were reported = unknown function	0
C	Membrane localization + Affects ion selectivity + Voltage-dependence of open probability + Deactivation at negative voltages of fast and slow gates +	11
C-D loop	Voltage-dependence of fast and slow gates + Link C-D is a loop that opens the vestibule, providing intracellular access * Directly involved in anion binding *	5
D	Contribute to the ClC-1 ion pathway + Part of the selectivity filter + Voltage-dependence of fast and slow gates + Channel opening kinetics at positive voltages and deactivation at negative voltages of fast and slow gates + Effects on rectification + Directly involved in anion binding *	10
D-E loop	NAD+ inhibition + Voltage-dependence of open probability +	2
E	Voltage-dependence of open probability +	9
E-F loop	Effect on current amplitude = specific function unknown	3
F	Voltage-dependence of open probability + Contribute to the ClC-1 ion pathway * Part of the selectivity filter * Directly involved in anion binding * Part of the fast gate (E232) *	11
F-G loop	NAD+ inhibition +	~5
G	Effects on ion selectivity and single channel conductance, suggesting a role in pore functioning + Effects on rectification + Voltage-dependence of fast and slow gating + Deactivation at negative voltages of fast and slow gates + Membrane localization +	12
H	Voltage-dependence of open probability + Part of the slow gate + Effects on rectification + Link between the two subunits * H-P interaction is probably necessary for channel assembly * Probably interact with CBS2 regarding their phosphorylation status during PKC modulation *	17
H-I loop	Voltage-dependence of open probability + Deactivation at negative voltages of slow gate + Effects on rectification +	3
I	Affects ion selectivity + Voltage-dependence of open probability + Link between the two subunits but not reported effects on slow gate kinetics * Probably interact with CBS2 regarding their phosphorylation status during PKC modulation *	22
I-J loop	Voltage-dependence of open probability + Probably part of the slow gate + Channel opening kinetics at positive voltages and deactivation at negative voltages of fast and slow gates +	19
J	Voltage-dependence of fast and slow gating + Conformational stability of the channel *	11
Helical stretch J-K	CBS interaction *	0
K	Poorly analyzed mutations reported = unknown function	7
Helical stretch K-L	Membrane localization + Voltage-dependence of open probability +	7
L	Channel opening kinetics at positive voltages and deactivation at negative voltages of fast and slow gates +	10
Helical stretch L-M	Voltage-dependence of open probability + Involved in trafficking + Deactivation at negative voltages of fast and slow gates +	7
M	Poorly analyzed mutations reported = unknown function	8
M-N loop	Voltage-dependence of the slow gate + Affects ion selectivity + Deactivation at negative voltages of fast and slow gates +	11
N	Voltage-dependence of fast and slow gates + Channel opening kinetics at positive voltages and deactivation at negative voltages of fast and slow gates + Contribute to the ClC-1 ion pathway + Part of the selectivity filter + Effects on rectification + Membrane localization + Directly involved in anion binding *	14
N-O loop	No analyzed mutations were reported = unknown function	1
O	Voltage-dependence of fast and slow gates + Channel opening kinetics at positive voltages and deactivation at negative voltages of fast and slow gates + Effects on rectification + Membrane localization + Conformational stability of the channel * Modulation of voltage dependency by pH *	14
O-P loop	Probably part of the slow gate +	1
P	Part of the slow gate + Link between the two subunits * H-P interaction is probably necessary for channel assembly * Role in binding zinc (possibly through the slow gate) *	5
P-Q loop	Voltage-dependence of open probability + Deactivation at negative voltages of the slow gate + Effects on rectification +	2
Q	Part of the slow gate + Voltage-dependence of fast and slow gates + Channel opening kinetics at positive voltages and deactivation at negative voltages of fast and slow gates + Effects on rectification + Link between the two subunits *	10
Q-R loop	Voltage-dependence of open probability +	2
R	Voltage-dependence of open probability + Contribute to the ClC-1 ion pathway * Part of the selectivity filter * Directly involved in anion binding * Helix R is close to helix A of the other monomer, possibly suggesting an interaction that might be important for slow gating *	1
C-terminal loops	Voltage-dependence of fast and slow gating + Involved in trafficking + Possibly involved in the pore structure + Modulation of the slow gate + ATP binding site * PKC phosphorylation site * Interacts with helix R linker, helix D, and intracellular H-I loop *	4 35 12
CBS1 CBS2	Modulation of the slow gate + Channel opening kinetics at positive voltages + Involved in trafficking * Involved in oligomerization *	20 19

+ Functions suggested by mutant characterizations. * Functions reported in the literature.

## Data Availability

Data was provided in supplementary tables.

## Supplementary Materials

The following supporting information can be downloaded at https://www.mdpi.com/article/10.3390/biomedicines11102622/s1. Supplementary Table S1: Mutations reported in the literature, nucleotide and amino acid (A.A.) change, protein localization regarding membrane and protein helices and loops, and inheritance; Supplementary Table S2: Described functional effects of mutations reported in the literature.
